# IGF‐1/SLC25A33 Maintains Blood‐Labyrinth Barrier Integrity by Suppressing TGF‐β/Smad2‐Mediated Pericyte Activation in Age‐Related Hearing Loss

**DOI:** 10.1111/acel.70712

**Published:** 2026-09-29

**Authors:** Rui Xu, Jia Tang, Huan Lin, Qi Li, Yajin Feng, Xiaorong Zhang, Xiaohong Hu, Jiacai Yang, Yuhong Li, Yang Bai

**Affiliations:** ^1^ Department of Otolaryngology, The First Affiliated Hospital Army Medical University Chongqing China; ^2^ Department of Neurology, The Second Affiliated Hospital Army Medical University Chongqing China; ^3^ Department of Cell Biology Army Medical University Chongqing China; ^4^ Institute of Burn Research The First Affiliated Hospital, Army Medical University Chongqing China

**Keywords:** ARHL, IGF‐1, pericyte, SLC25A33, TGF‐β/Smad signaling

## Abstract

Age‐related hearing loss (ARHL) is a prevalent sensory disorder in the elderly, yet the molecular mechanisms underlying cochlear aging remain incompletely understood. While insulin‐like growth factor 1 (IGF‐1) has been implicated in ARHL, its role in the stria vascularis, particularly in pericyte‐mediated blood‐labyrinth barrier (BLB) regulation, is poorly defined. We performed transcriptomic analysis of FACS‐purified pericytes from young and aged mouse stria vascularis. Functional studies were conducted using primary pericyte cultures, lentiviral gene modulation, and AAV‐mediated in vivo delivery. Auditory function was assessed by auditory brainstem response (ABR) and distortion product otoacoustic emissions (DPOAE). Molecular changes were evaluated via qPCR, western blot, immunofluorescence, flow cytometry, and transmission electron microscopy (TEM). Transcriptomics revealed significant downregulation of *Igf1* and *Slc25a33* in aged pericytes, with enrichment of the TGF‐β signaling pathway. IGF‐1 deficiency suppressed SLC25A33 expression in vitro, leading to mitochondrial ROS (mtROS) accumulation, activation of TGF‐β/Smad2 signaling, and pericyte phenotypic switching, characterized by increased α‐SMA and decreased E‐cadherin. In vivo, suppression of IGF‐1 or SLC25A33 activated TGF‐β/Smad2 signaling in the stria vascularis, disrupted BLB ultrastructure, and exacerbated hearing loss. Conversely, exogenous IGF‐1 or SLC25A33 overexpression attenuated these effects and partially restored auditory function. Our findings identify a novel IGF‐1/SLC25A33–TGF‐β/Smad2 axis that regulates pericyte activation and BLB integrity in the aging cochlea. This pathway provides mechanistic insight into ARHL pathogenesis and highlights a potential therapeutic target for preserving hearing in older adults.

## Graphical abstract

IGF‐1/SLC25A33–TGF‐β/Smad2 axis can regulate pericyte activation and BLB integrity, which may be a potential therapeutic target for preserving hearing in ARHL.

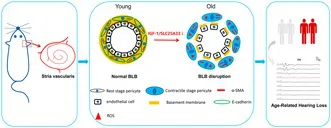

## 1 Introduction

Age‐related hearing loss (ARHL), or presbycusis, is the third most prevalent chronic condition in older adults and affects nearly half of individuals as they age (Celaya et al. 2021; Ege et al. 2024). Beyond pure‐tone threshold elevation, ARHL is characterized by impaired speech discrimination and poor sound localization, deficits that are particularly pronounced in noisy environments. Unmanaged ARHL has been linked to increased risks of social withdrawal, depression, and cognitive decline, ultimately contributing to a higher incidence of dementia (Magarinos et al. 2015). With global populations aging rapidly, the public health burden of ARHL is expected to rise substantially in the coming decades (Rodriguez‐de la Rosa et al. 2017).

The onset and progression of ARHL vary widely across individuals, suggesting a multifactorial etiology involving both intrinsic and extrinsic factors. Genetic predisposition, noise exposure, ototoxic drugs, and metabolic stress all appear to contribute to cochlear aging. Despite growing interest, the molecular and cellular mechanisms driving age‐related cochlear degeneration remain incompletely understood (Affortit et al. 2021).

Genetic studies have identified several candidate genes associated with ARHL susceptibility (Affortit et al. 2021; Almalki 2024; Fransen et al. 2015; Koffler et al. 2015; Op de Beeck et al. 2011; Salminen and Kaarniranta 2010). Among them, insulin‐like growth factor 1 (IGF‐1) has emerged as a key regulator of hearing function. IGF‐1 is crucial not only for embryonic and postnatal development of the inner ear but also for lifelong maintenance of auditory homeostasis in both peripheral and central pathways (Riquelme et al. 2010; Tafra et al. 2014; Varela‐Nieto et al. 2013). IGF‐1 expression peaks during late cochlear maturation, declines after birth, and gradually decreases with aging, though it remains detectable throughout life (Murillo‐Cuesta et al. 2011; Okano et al. 2011; Riva et al. 2007). Notably, reduced circulating or tissue‐specific IGF‐1 levels correlate with accelerated hearing loss in human populations (Lassale et al. 2017), underscoring its relevance to ARHL pathogenesis.

Within the cochlea, IGF‐1 is predominantly expressed in spiral ganglion neurons and the stria vascularis. Previous research on IGF‐1 in hearing loss has largely centered on its neuroprotective effects and roles in mitigating oxidative stress, mitochondrial dysfunction, and excitotoxicity—processes that drive degeneration of hair cells (HCs) and spiral ganglion neurons (SGNs) (Bao and Ohlemiller 2010; Bowl and Dawson 2015; Riquelme et al. 2010). While the stria vascularis is also a major site of IGF‐1 expression (Garcia‐Mato et al. 2021), its functional involvement in ARHL has received limited attention. To date, only Riquelme et al. have reported progressive stria vascularis atrophy in *Igf1* ^−/−^ mice, highlighting a potential structural role. Nevertheless, the mechanistic link between IGF‐1 deficiency and stria vascularis dysfunction in aging remains unclear.

SLC25A33 (also known as PNC1), a mitochondrial nucleotide carrier, is an IGF‐1/IGF‐1R‐responsive protein that sustains mitochondrial function. Knockdown of SLC25A33 has been shown to impair oxidative phosphorylation (OXPHOS), induce mitochondrial reactive oxygen species (mtROS) leakage from the electron transport chain, and trigger a robust, ROS‐dependent epithelial‐to‐mesenchymal transition (EMT)‐like phenotypic switch in MCF‐7 breast cancer cells (Favre et al. 2010; Floyd et al. 2007; Lyons et al. 2017), While SLC25A33 overexpression is sufficient to prevent TGF‐β‐induced EMT (Favre et al. 2010). Given the high metabolic demand and mitochondrial density in cochlear tissues, we hypothesized that age‐related downregulation of IGF‐1 suppresses SLC25A33 activity in stria vascularis pericytes, promoting their transition toward a pro‐fibrotic, contractile phenotype via TGF‐β/Smad signaling. This pericyte activation would compromise the integrity of the blood‐labyrinth barrier (BLB) (Yao et al. 2014), initiate microvascular dysfunction, and ultimately contribute to cochlear aging and ARHL.

In this study, we demonstrate that aging‐associated IGF‐1 deficiency directly downregulates SLC25A33 in pericytes, activating the TGF‐β/Smad pathway and driving pericyte phenotypic switching from a quiescent, stabilizing state to an activated, contractile myofibroblast‐like state. This leads to morphological disruption of the BLB and cochlear dysfunction. Our findings reveal a novel IGF‐1/SLC25A33–TGF‐β/Smad axis in pericyte‐mediated BLB regulation, providing new mechanistic insights into ARHL and identifying a potential therapeutic target for preserving hearing in aging.

## 2 Methods

### 2.1 Animals

Specific pathogen‐free (SPF) wild‐type C57BL/6J mice were obtained from the Laboratory Animal Center of the Army Medical University. Young (3‐month‐old) and aged (18‐month‐old) male and female mice were used in this study. All animal procedures were approved by the Animal Ethics Committee of the Army Medical University (AMUWEC20224078) and conducted in accordance with the ARRIVE guidelines.

### 2.2 Cell Culture and Treatment

1. Primary cochlear pericytes were isolated and cultured according to a previously described protocol (Neng et al. 2013) with minor modifications.
  - Dissection of stria vascularis: postnatal Day 12–16 (P12‐P16) C57BL/6J mice were anesthetized and euthanized. The mice were decapitated rapidly, and the auditory bullae were immediately dissected and placed in prechilled fresh artificial perilymph solution (Biochemazone, Canada). The stria vascularis was gently separated from the cochlear spiral ligament in ice‐cold perilymph, and the entire isolation procedure was completed within 1 h to minimize cell damage.
  - Cell culture and cell type selection: the isolated stria vascularis tissues were rinsed in cold perilymph solution with gentle shaking for 10 min, then transferred to collagen I‐coated dishes containing low‐glucose Dulbecco's Modified Eagle's Medium (DMEM, iCell, China) culture medium. Under a dissecting microscope, the tissues were minced into small fragments of 0.15–0.20 mm^3^ using sterile ophthalmic tweezers. The tissue fragments were incubated at 37°C with 5% CO_2_ in pericyte‐selective culture medium. The pericyte‐specific culture medium was prepared with low‐glucose DMEM (1 g/L glucose) supplemented with 10% fetal bovine serum (FBS, Hyclone, USA), 1% penicillin–streptomycin (Biochannel, China), and 100 nM pigment epithelium‐derived factor (PEDF, Sigma, Germany). Strict control of glucose concentration and PEDF supplementation effectively promoted pericyte proliferation and inhibited the growth of endothelial cells, ensuring the purity of primary pericytes.
  - Medium was partially replaced (50%) at 48 h and on Day 3. Cellular outgrowth typically emerged within 7‐14 days. Primary cultured pericytes were used for subsequent immunofluorescence staining of IGF‐1, SLC25A33, and IGF‐1R.
2. Immortalized Human Brain Vascular Pericytes (HBVPs)
  HBVPs (catalog No. ZQY006) were purchased from Shanghai Zhong Qiao Xin Zhou Biotechnology Co. Ltd. Cells were incubated at 37°C in a humidified atmosphere consisting of 95% air and 5% CO_2_ using customized complete medium. The medium was composed of 500 mL basal medium supplemented with 10 mL FBS, 5 mL cell growth factors, and 5 mL penicillin–streptomycin solution (dedicated medium catalog No. ZQ‐1321). For experimental treatments, cultures were exposed to either 10 μM NVP‐AEW541 (IGF‐1R inhibitor; MCE, USA) or 200 ng/mL recombinant mouse IGF‐1 (791‐MG, R&D Systems, USA).

### 2.3 Transcriptome Sequencing and Pathway Enrichment Analysis

Primary pericytes were isolated from microdissected stria vascularis of young and aged mice using fluorescence‐activated cell sorting (FACS) with specific surface markers: PE Anti‐PDGFR beta antibody (Abcam, ab93534) and Anti‐CD31 antibody (R&D systems, AF3628). Details of the gating strategy for isolating pericytes are presented in Figure S1. Purified cells were sent to Knorigene Technologies Co. Ltd. (Chongqing, China) for transcriptome sequencing based on the Smart‐seq2 protocol. Briefly, sorted cells were lysed, and total RNA was precipitated with 80% ethanol. First‐strand cDNA synthesis was performed using an Oligo (dT) primer (5′‐AAGCAGTGGTATCAACGCAGAGTACT_30_VN‐3′) and SuperScript II Reverse Transcriptase (Invitrogen, cat. no. 18064‐014). A template‐switching oligo (5′‐AAGCAGTGGTATCAACGCAGAGTACATrGrG+G‐3′) was used to enable full‐length cDNA amplification via ISPCR (5′‐AAGCAGTGGTATCAACGCAGAGT‐3′). Amplified cDNA was normalized and subjected to library construction using the TruePrep DNA Library Prep Kit V2 for Illumina (Vazyme Biotech), followed by clustering and paired‐end sequencing on an Illumina platform. Differentially expressed genes were identified using DESeq2. Disease Ontology (DO) and Gene Ontology (GO) enrichment analyses were conducted using the ReactomePA and clusterProfiler packages, respectively. Reactome and Kyoto Encyclopedia of Genes and Genomes (KEGG) pathway enrichment analysis were performed to identify signaling pathways associated with aging. Protein‐protein interaction network (PPI) analysis was performed using STRINGdb package.

### 2.4 Lentiviral Transduction for SLC25A33 Modulation

HBVPs were seeded at 1 × 10^5^ cells/cm^2^ in 12‐well plates and allowed to adhere overnight. Lentiviruses encoding *Slc25a33* overexpression (LV‐*Slc25a33*‐OE), shRNA‐mediated knockdown (LV‐*Slc25a33*‐shRNA), or empty vector control (LV‐NC) were obtained from Tsingke BiotechnologyCo. Ltd. (Beijing, China). The coding sequences of the insert in LV‐*Slc25A33*‐OE and LV‐*Slc25A33*‐shRNA are provided in Supporting Information. Transduction was performed at multiplicity of Infection (MOI) = 30 in medium containing 2% FBS and 8 μg/mL polybrene. After 24 h, fresh complete medium was added. Puromycin selection (10 μg/mL) began 48 h post‐transduction and continued for 5‐7 days until stable polyclonal populations were established.

### 2.5 mtROS Assay

mtROS levels were measured using MitoSOX Red (Beyotime, China). HBVPs (1 × 10^7^ cells/mL) were incubated with diluted dye at 37°C for 60 min in the dark. Cells were washed with PBS, resuspended, and kept on ice protected from light. Fluorescence was analyzed by flow cytometry (excitation: 396 nm; emission: 580 nm) with ≥ 10,000 events collected per sample. Data were processed using FlowJo software.

### 2.6 Real‐Time Quantitative PCR

Total RNA was extracted from HBVPs or FACS‐purified pericytes isolated from stria vascularis, using the RNeasy Mini Kit (QIAGEN, Germany) and quantified spectrophotometrically (DU 800, Beckman Coulter). cDNA was synthesized using a First Strand cDNA Synthesis Kit (TOYOBO, Japan). qPCR was performed with SYBR Green PCR Master Mix (TOYOBO) on an ABI PRISM 7500 system under the following cycling conditions: 95°C for 2 min; 50 cycles of 95°C for 15 s, 60°C for 15 s, and 72°C for 32 s. Primer sequences are provided in Table S1. Gene expression was calculated with *Gapdh* as the reference gene.

### 2.7 Western Blot Analysis

HBVPs were lysed in ice‐cold Radio Immunoprecipitation Assay (RIPA) lysis buffer containing protease and phosphatase inhibitors (Beyotime, China). Protein concentration was determined using a Bicinchoninic Acid (BCA) assay (Thermo Fisher, USA). Equal amounts (20 μg) of protein were separated on 10% Sodium Dodecyl Sulfate‐Polyacrylamide Gel Electrophoresis (SDS‐PAGE) gels (80 V for 30 min, then 100 V for 1.5 h) and transferred to 0.22 μm Polyvinylidene Fluoride (PVDF) membranes. Membranes were blocked in 5% non‐fat milk for 1 h and incubated overnight at 4°C with primary antibodies against: E‐cadherin (Bioworld, BS80070, 1:500), α‐SMA (Bioss, bs‐10196R, 1:2000), TGFβ1 (Bioworld, BS80030, 1:500), Smad2 (Bioworld, BS1425, 1:500), Smad3 (Bioworld, BS1426, 1:500), Smad4 (Bioworld, BS45204, 1:1000), p‐Smad2 (Bioworld, MB11895, 1:500), p‐Smad3 (Bioworld, BZ16381, 1:500), and GAPDH (Cell Signaling Technology, 1:5000). After washing, membranes were incubated with Horseradish Peroxidase (HRP)‐conjugated secondary antibodies (Beyotime, 1:2000) for 1 h at room temperature. Signals were detected using enhanced chemiluminescence (Beyotime) and imaged with a ChemiDoc XRS system (Bio‐Rad, USA).

### 2.8 Construction of AAV‐ie Vectors for SLC25A33 Modulation

The coding sequence of *Slc25a33* (NM_027460.2, 963 bp) was cloned into an Adeno‐Associated Virus (AAV)‐ie vector using *Escherichia coli* Restriction Enzyme (EcoR) I and EcoRV restriction sites. The construct included a C‐terminal 3 × FLAG tag, Cytomegalovirus (CMV) promoter, Elongation Factor 1 alpha (EF1α)‐zsGreen1 reporter, WPRE element, and flanking AAV2 Inverted Terminal Repeat (ITRs), and was designated AAV‐ie‐*Slc25a33*. For knockdown, a U6‐driven shRNA targeting *Slc25a33* was inserted into a dual‐promoter AAV‐ie vector co‐expressing EGFP under the CMV promoter (AAV‐ie‐sh*Slc25a33*). Sanger sequencing was applied to validate all recombinant constructs. The AAV‐ie‐*Slc25a33* vector was sequenced with CMV‐F (5′‐CGCAAATGGGCGGTAGGCGTG‐3′) and EF1α‐SEQR (5′‐CCAACTTCTCGGGGACTGTG‐3′). The AAV‐ie‐sh*Slc25a33* vector was verified using U6‐specific sequencing primers (5′‐GCTGGAATTTCCGAAACTATA‐3′).

### 2.9 Virus Production

AAV particles (AAV‐ie‐*Slc25a33* and AAV‐ie‐sh*Slc25a33*) were produced by transient transfection of HEK293T cells with vector, capsid, and helper plasmids. Viruses were packaged and purified by OBiO Technology Corp. Ltd. (Shanghai, China). Genome titers were determined by qPCR and stored at −80°C until use.

### 2.10 In Vivo Delivery of IGF‐1, Inhibitor, or AAV Vectors via Round Window Membrane

Mice were anesthetized with Delivector Avertin (Tribromoethanol, TBE, 20 μL/g). The postauricular region was shaved and disinfected with 10% povidone‐iodine. Under an operating microscope, a small hole was made in the tympanic bulla to expose the round window membrane (RWM). A glass micropipette connected to a nanoliter microinjection system was used to penetrate the RWM. After stabilization (no perilymph leakage), 2 μL of agent was injected at 5 nL/s. For pharmacological studies: 10 μM NVP‐AEW541 or 200 ng/mL IGF‐1 was delivered; behavioral and molecular analyses were performed 2 weeks later. For viral studies: AAV‐ie‐*Slc25a33* (4.71 × 10^12^ vg/mL), AAV‐ie‐sh*Slc25a33* (2.88 × 10^12^ vg/mL), or control vector was administered; experiments were conducted 4 weeks post‐injection.

### 2.11 Immunofluorescence Staining

For in vitro staining, primary pericytes were fixed with 4% paraformaldehyde (PFA), permeabilized, and blocked with blocking buffer (P0102, Beyotime). For in vivo, cochleae were harvested and fixed in 4% PFA at 4°C overnight. Apical openings facilitated perfusion. Samples were decalcified in 10% EDTA for 3 days, embedded, and sectioned. Sections were blocked in 10% goat serum + 0.3% Triton X‐100 in PBS for 1 h. Primary antibodies were applied overnight at 4°C: IGF‐1 (NOVUS, SPM406, 1:200), SLC25A33 (Bioss, bs‐21231R, 1:200), PDGFRβ (CST, #3169, 1:200), E‐cadherin (CST, #14472, 1:200), α‐SMA (Bioss, bs‐10196R, 1:200), TGFβ1 (Bioworld, BS80030, 1:50), p‐Smad2 (Bioworld, bs‐3419R, 1:500). Secondary antibodies: Alexa Fluor 555 anti‐mouse (Invitrogen, A‐21422, 1:1000), Alexa Fluor 647 anti‐rabbit (Invitrogen, A‐31573, 1:1000). Nuclei were stained with DAPI (Beyotime, C1002). Slides were mounted with VectorShield (Vectorlabs) and imaged by confocal microscopy.

Five typical sections from five individual mice were analyzed per group. The detailed procedure for fluorescence intensity analysis was as follows: (1) First, the region of the stria vascularis was defined and selected as the area of interest (AOI) by two experienced pathologists; (2) The cumulative optical density (OD) value of specific protein within the stria vascularis region was measured; (3) The area of the stria vascularis (i.e., AOI) was measured; (4) The mean fluorescence intensity (MFI) was calculated as follows: MFI = cumulative OD value/area of the stria vascularis; (5) The relative MFI was calculated using the average MFI of the young control group as the baseline.

### 2.12 Auditory Function Testing

Auditory brainstem response (ABR) and distortion product otoacoustic emissions (DPOAE) were recorded using the TDT BioSigRP system (Tucker‐Davis Technologies) in a sound‐attenuating chamber. Mice were anesthetized with tribromoethanol (20 μL/g, i.p.) and maintained on a heating pad. Subcutaneous electrodes were placed at the vertex, ventral edge of the pinna, and tail (ground).

ABR: Click stimuli and tone bursts (4, 8, 12, 16, 32 kHz) were presented. Bandpass filter: 300–3000 Hz; 512 sweeps averaged; sweep duration: 10 ms. Stimulus intensity started at 90 dB SPL, decreased in 10‐dB steps, then 5‐dB near threshold. ABR threshold was defined as the lowest intensity yielding a reproducible Wave II.

DPOAE: f_1_ and f_2_ tones (f_2_/f_1_ = 1.2) were used. Maximum level: 80 dB SPL, reduced in 5‐dB steps. DPOAE threshold was the lowest intensity producing a detectable f_1_–f_2_ response. Noise floor remained below 0 dB across frequencies.

### 2.13 Transmission Electron Microscopy (TEM)

After auditory testing, temporal bones were dissected and fixed in glutaraldehyde‐based fixative (Servicebio, G1102). Samples were decalcified in Ethylenediaminetetraacetic acid (EDTA) solution (Servicebio, G1105) for 3 days on a shaker. Post‐fixation: 1% OsO_4_ in 0.1 M phosphate buffer (pH 7.4) for 2 h. Washes: three times in PB (15 min each). Dehydration: graded ethanol series (30%–100%), then acetone. Infiltration and embedding in EMBed 812 resin. Ultrathin sections (80 nm) were cut, collected on grids, and stained with 2% uranyl acetate and 2.6% lead citrate (8 min each). Grids were dried overnight and examined under a transmission electron microscope (HT7800, Hitachi, Japan).

### 2.14 Statistical Analysis

All experiments included appropriate controls. Sample sizes were estimated based on pilot data using power analysis (Sample Size Calculator). A blinded design was implemented to minimize bias. Data are presented as mean ± SEM. Statistical analyses were performed using GraphPad Prism 9. Two‐group comparisons used unpaired two‐tailed *t*‐tests. After data normality and homogeneity of variance were tested and confirmed, multiple groups were analyzed by one‐way ANOVA, followed by Bonferroni's test. Multiple comparisons for auditory threshold measurements were performed using the Kruskal‐Wallis test followed by Dunn's post hoc test. Statistical significance was set at *p <* 0.05.

## 3 Results

### 3.1 Transcriptomic Analysis Identified IGF‐1/SLC25A33 Dysregulation and TGF‐β Pathway Activation in Aged Pericytes, Which Might be Associated With Strial Degeneration and ARHL

Compared to young mice, aged animals exhibited significant hearing impairment. ABR testing revealed that 85% of old mice failed to respond to stimuli below 70 dB SPL (Table S2). Click‐evoked ABR thresholds were markedly elevated in aged mice, and frequency‐specific thresholds were significantly increased across all tested frequencies. Consistent with sensory dysfunction, DPOAE thresholds were significantly higher in old mice at 17.44 kHz, indicating cochlear amplifier deficits (Figure 1A–D).

**FIGURE 1:**
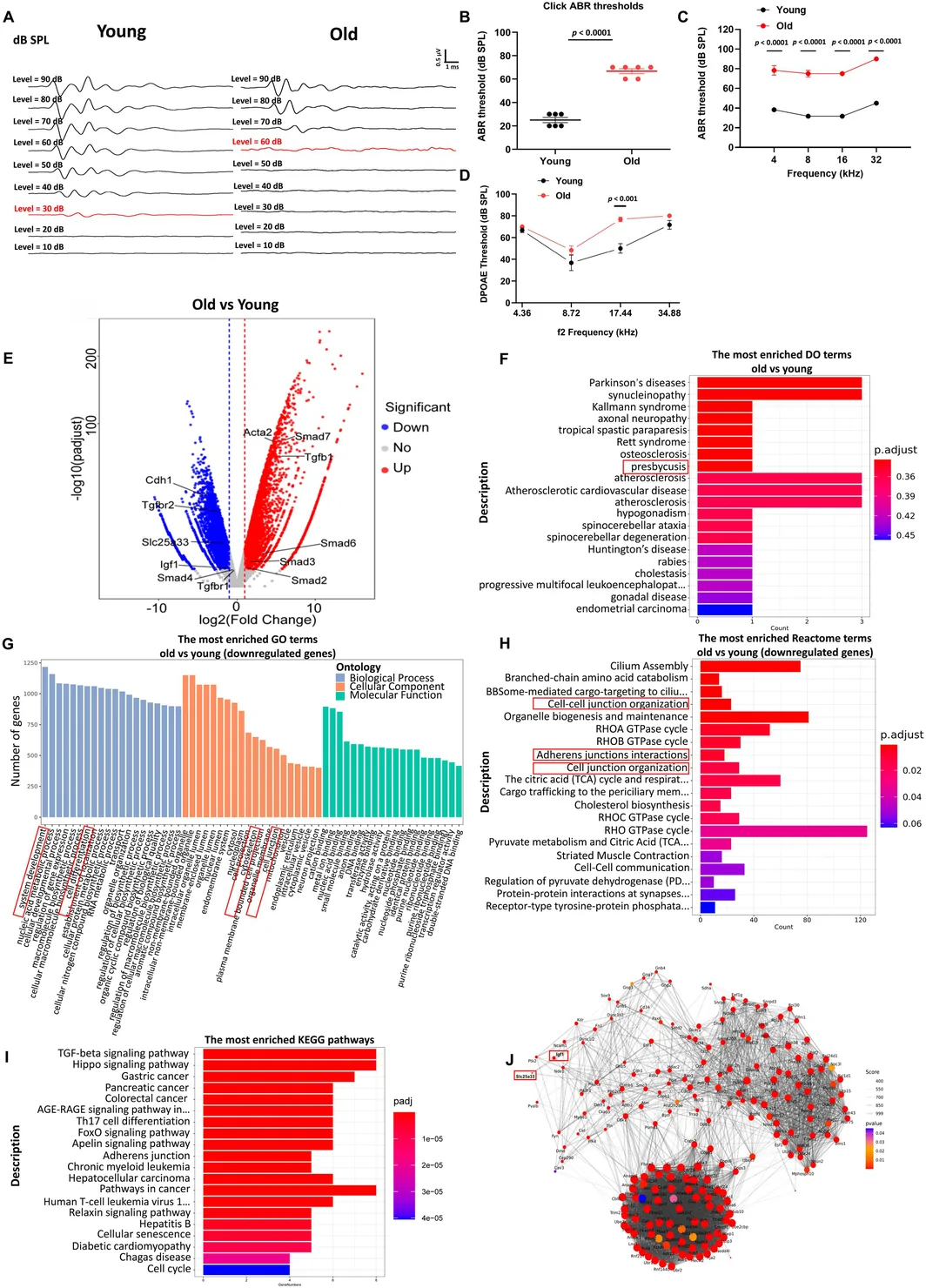
Transcriptomic analysis identifies IGF‐1/SLC25A33 dysregulation and TGF‐β pathway activation in aged pericytes, which may be associated with ARHL. (A) Representative click‐evoked ABR waveforms showing a threshold of 30 dB SPL (red trace, left) in a young mouse versus 60 dB SPL (red trace, right) in an aged mouse. (B) Click‐evoked ABR thresholds are significantly elevated in old mice compared to young controls. (C) Frequency‐specific ABR thresholds are markedly increased across all tested frequencies (4–32 kHz) in aged mice. (D) DPOAE thresholds are significantly higher in old mice at F_2_ = 17.44 kHz (*n* = 6 mice). (E) RNA sequencing of FACS‐purified pericytes from young and aged stria vascularis reveals significant downregulation of *Igf1* and *Slc25a33* mRNA in aged mice (*n* = 6 mice). (F) The most enriched DO terms for the differentially expressed genes when old vs. young. (G) The most enriched GO terms for the downregulated genes when old versus young. (H) The most enriched Reactome terms for the downregulated genes when old versus young. (I) KEGG pathway enrichment analysis highlights the TGF‐β signaling pathway as one of the most differentially activated pathways. (J) Gene–gene interaction network for the downregulated genes when old versus young.

To explore molecular changes underlying strial degeneration, we performed transcriptome sequencing on FACS‐purified pericytes isolated from stria vascularis of young and aged mice (Figure S1). This analysis revealed significant downregulation of both *Igf1* and *Slc25a33* mRNA in aged pericytes (Figure 1E, Tables S3–S5). Among the DO enrichments, presbycusis was identified as one of the most enriched diseases (Figure 1F). In GO analysis, system development and cell differentiation were found to be the most remarkable BP annotations, and cytoskeleton and cell junction were the most remarkable CC annotations (Figure 1G). The most enriched Reactome terms were cell–cell junction organization, adherens junctions interactions and cell junction organization (Figure 1H). And KEGG pathway enrichment identified the TGF‐β signaling pathway as one of the most differentially activated pathways in aged samples (Figure 1I). The gene interactions involving *Igf1* and *Slc25a33 were* presented in Figure 1J.

### 3.2 Downregulation of IGF‐1 and SLC25A33 Were Confirmed in the Aged Stria Vascularis. In Vitro Studies Revealed IGF‐1 Regulated SLC25A33 Expression

In vivo validation using FACS‐purified pericytes isolated from stria vascularis of young and aged mice confirmed downregulation of *Igf1* and *Slc25a33* mRNA expression in aged pericytes (Figure 2A,B). Immunofluorescence staining confirmed co‐expression of IGF‐1 and SLC25A33 within the stria vascularis (ribbon‐like structure) and revealed a significant age‐dependent downregulation of both proteins (Figure 2C–E), suggesting their potential role in strial aging. More importantly, although age‐dependent alteration of IGF‐1 and SLC25A33 was also detected in endothelial cells and other cochlear cell types within the stria vascularis, such changes markedly occurred in pericytes (Figure 2C, indicated by arrows).

**FIGURE 2:**
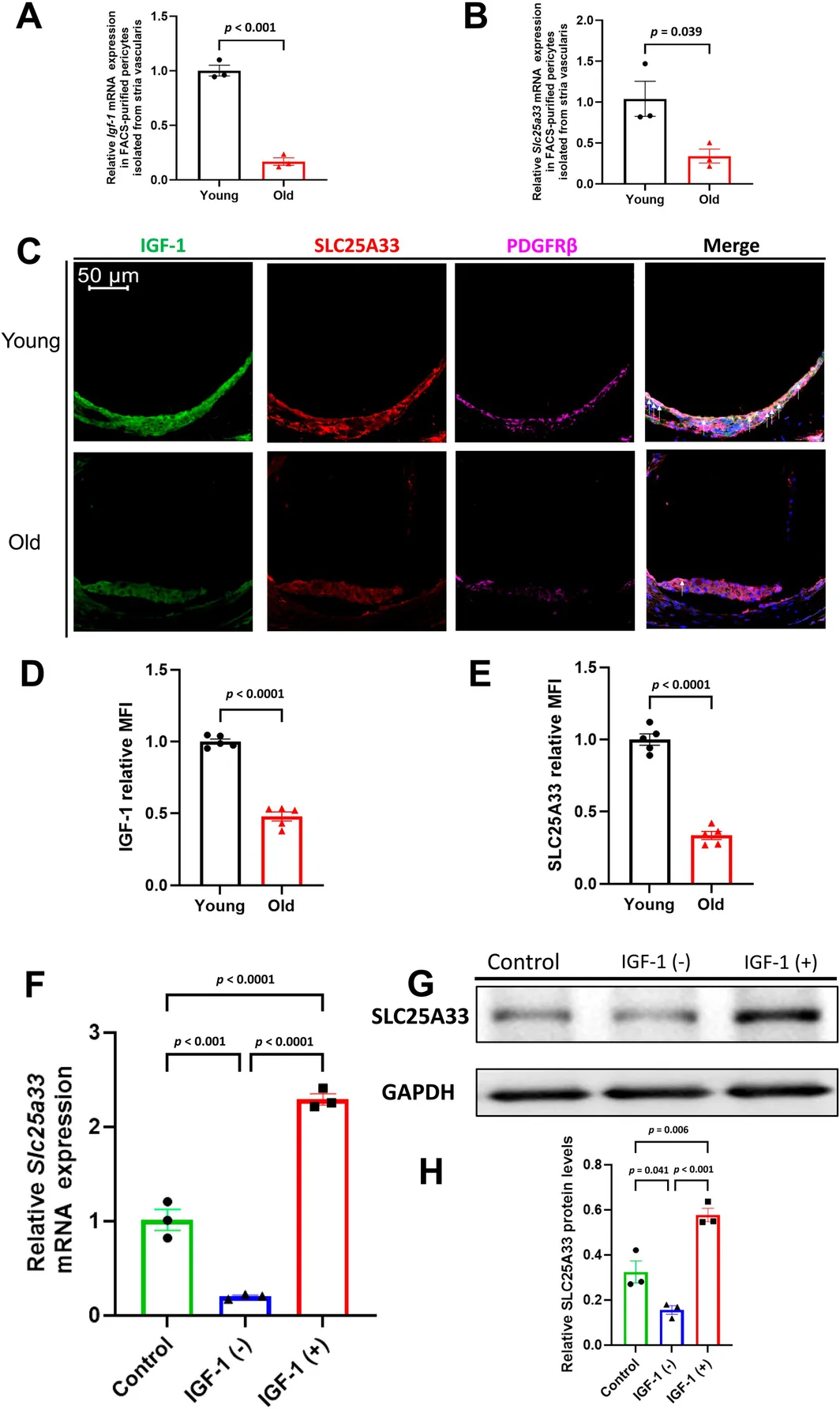
Downregulation of IGF‐1 and SLC25A33 are confirmed in the aged stria vascularis. In vitro studies reveal IGF‐1 regulates SLC25A33 expression. (A, B) qPCR shows downregulation of *Igf1* and *Slc25a33* mRNA expression in aged pericytes (*n* = 3 mice); (C) Immunofluorescence staining confirms colocalization of IGF‐1 (green), SLC25A33 (red) and PDGFRβ (purple, pericyte marker) in the stria vascularis. Age‐dependent alteration of IGF‐1 and SLC25A33 were markedly occurred in pericytes, as indicated by arrows. (D‐E) Quantitative analysis reveals significant downregulation of both IGF‐1 and SLC25A33 fluorescence intensity in aged mice relative to young controls (*n* = 5, five typical sections from five individual mice were analyzed per group). (F) qPCR shows that inhibition of IGF‐1 signaling (via NVP‐AEW541) significantly reduces *Slc25a33* mRNA levels. (G) Representative Western blot of SLC25A33 protein expression. (H) Densitometric quantification demonstrates reduced SLC25A33 protein upon IGF‐1 inhibition. Control: HBVPs without treatment; IGF‐1 (−): HBVPs treated with NVP‐AEW541; IGF‐1 (+): Cells treated with recombinant IGF‐1. Data are mean ± SEM; *n* = 3 mice.

In vitro validation using primary pericyte cultures derived from postnatal mice (Figure S2) further confirmed these findings. Immunofluorescence demonstrated co‐localization of IGF‐1, IGF‐1R, and SLC25A33 in cultured pericytes (Figure S3A); however, no significant difference in *Igf1r* mRNA levels was detected between FACS‐purified pericytes from the stria vascularis of young and aged mice (Figure S3B). Furthermore, inhibition of IGF‐1 signaling with NVP‐AEW541 significantly reduced both *Slc25a33* mRNA and protein levels, while recombinant IGF‐1 treatment upregulated SLC25A33 expression (Figure 2F–H), establishing a direct regulatory link between IGF‐1 and SLC25A33. Notably, modulation of IGF‐1 signaling in cultured pericytes directly regulated SLC25A33 expression, whereas no such effect was observed in endothelial cells (Figure S4).

### 3.3 SLC25A33 Deficiency Induces mtROS Production and Drives Pericyte Differentiation

Given the known mitochondrial function of SLC25A33, we assessed mtROS levels using MitoSOX Red. Suppression of *Slc25a33* (via shRNA, Figure S5A) significantly increased mtROS production compared to control and overexpression groups (Figure 3A,B). We next evaluated pericyte phenotypic switching, a hallmark of microvascular dysfunction. α‐Smooth muscle actin (α‐SMA), a marker of myofibroblast‐like differentiation, was significantly upregulated at both mRNA and protein levels in SLC25A33‐deficient pericytes (Figure 3C,E,F). Conversely, E‐cadherin, a junctional protein associated with quiescent pericytes—was markedly downregulated upon *Slc25a33* knockdown (Figure 3D,E,G), indicating loss of barrier‐stabilizing properties.

**FIGURE 3:**
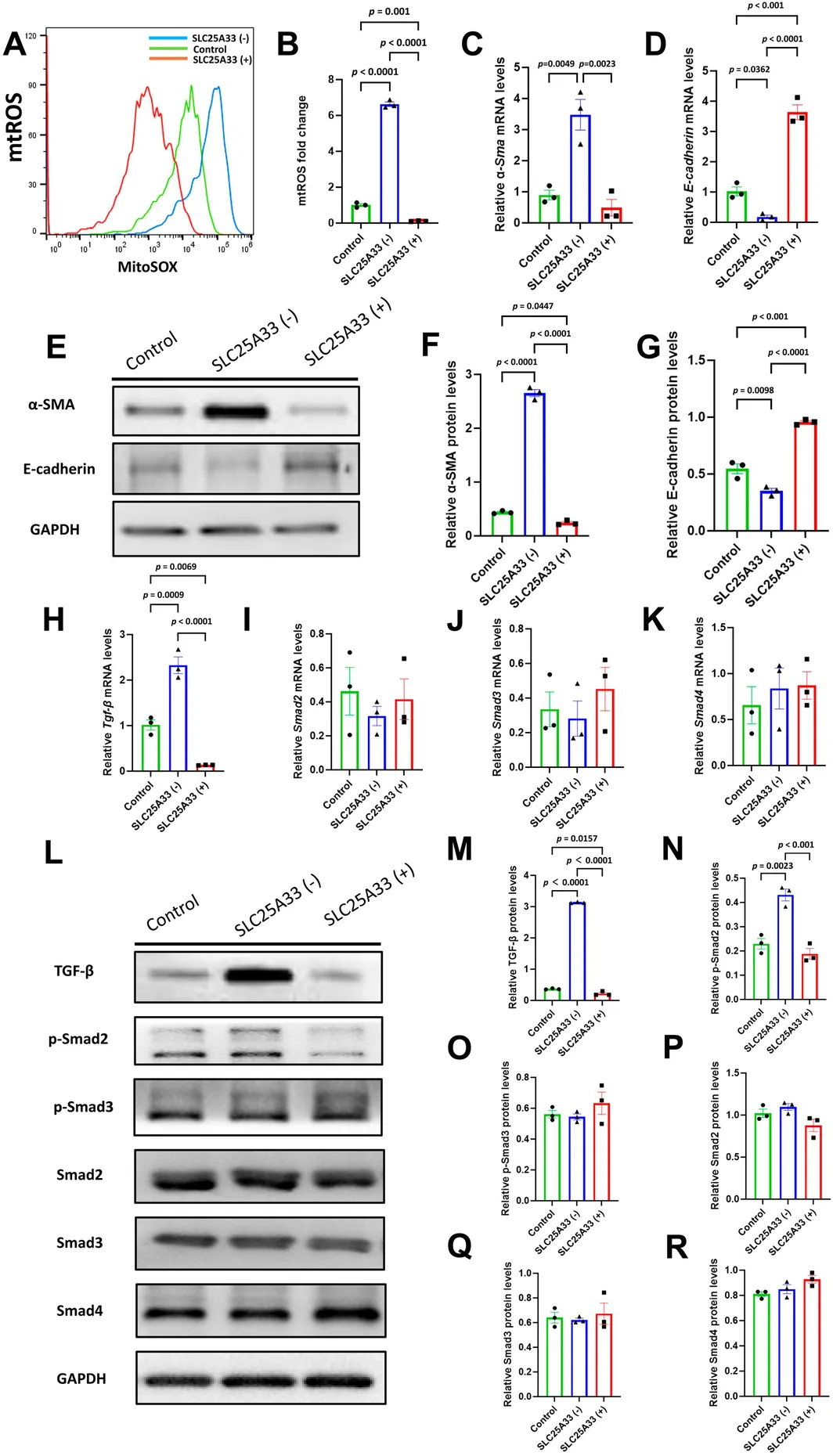
SLC25A33 deficiency induces pericyte differentiation via mitochondrial ROS accumulation, which were regulated by the TGF‐β/Smad2 signaling pathway. (A) Flow cytometry analysis using MitoSOX Red shows increased mtROS production in SLC25A33‐suppressed HBVPs compared to control and SLC25A33‐overexpressing groups. (B) Quantification of mtROS fluorescence intensity confirms this increase. (C, D) qPCR analysis reveals upregulated *α‐Sma* and downregulated *E‐cadherin* mRNA levels in *Slc25a33*‐knockdown HBVPs. (E) Representative Western blot images of α‐SMA and E‐cadherin protein expression. (F, G) Densitometric quantification shows increased α‐SMA and decreased E‐cadherin protein levels. (H) qPCR analysis shows upregulated *Tgfb1* mRNA in SLC25A33‐deficient HBVPs. (I–K) No significant changes are observed in *Smad2*, *Smad3*, or *Smad4* mRNA levels among groups. (L) Representative western blots of TGF‐β, p‐Smad2, Smad2, p‐Smad3, Smad3, Smad4, and GAPDH. (M) TGF‐β protein levels are significantly elevated in the knockdown group. (N) Phosphorylated Smad2 (p‐Smad2) is markedly increased, indicating pathway activation. (O–R) No significant differences are detected in p‐Smad3, total Smad2, Smad3, or Smad4 protein expression. Control: HBVPs without treatment; SLC25A33 (−): HBVPs treated with LV‐*Slc25a33*‐shRNA; SLC25A33 (+): HBVPs treated with LV‐*Slc25a33*‐OE. Data are mean ± SEM; *n* = 3.

### 3.4 The TGF‐β/Smad2 Signaling Pathway Mediates SLC25A33‐Regulated Pericyte Differentiation

Since TGF‐β signaling was enriched in our transcriptomic data and is a well‐known inducer of α‐SMA, we examined its activation status. qPCR and Western blot analyses showed that *Tgf‐β* mRNA and protein levels were significantly elevated in *Slc25a33*‐suppressed pericytes (Figure 3H,L,M). Although total Smad2/3/4 expression remained unchanged (Figure 3I–K,P–R), phosphorylation of Smad2 (p‐Smad2) was robustly increased in the knockdown group (Figure 3L,N), indicating selective activation of the canonical TGF‐β/Smad2 axis. No significant change in p‐Smad3 was observed (Figure 3L,O), supporting pathway specificity. Besides, treatment of *Slc25a33*‐suppressed cells with MitoTEMPO diminished elevated mtROS and decreased TGF‐β, p‐Smad2, and α‐SMA expression, verifying mitochondrial ROS acted upstream of TGF‐β activation (Figure S6).

We further manipulated the TGF‐β pathway in SLC25A33‐deficient pericytes using the TGF‐β receptor inhibitor LY2109761 (Figure S7A–D). The results demonstrated that inhibition of the TGF‐β signaling pathway in SLC25A33‐deficient pericytes significantly downregulated the protein levels of p‐Smad2 and α‐SMA, while increasing E‐cadherin expression. Collectively, these findings verified that SLC25A33 deficiency triggers TGF‐β pathway activation and consequently promotes pericyte differentiation.

### 3.5 In Vivo Modulation of IGF‐1 Regulates SLC25A33 Expression in the Aging Stria Vascularis

To validate these findings in vivo, we delivered IGF‐1 or its inhibitor (NVP‐AEW541) via round window membrane microinjection into aged mice. Immunofluorescence revealed that exogenous IGF‐1 supplementation significantly enhanced SLC25A33 expression in the stria vascularis, whereas IGF‐1 inhibition further reduced it (Figure 4A–C). qPCR confirmed exogenous IGF‐1 supplementation upregulated *Slc25a33* mRNA levels, whereas IGF‐1 inhibition decreased its expression (Figure 4D). As expected, both IGF‐1 and SLC25A33 levels were lower in aged mice than in young mice, confirming age‐related decline.

**FIGURE 4:**
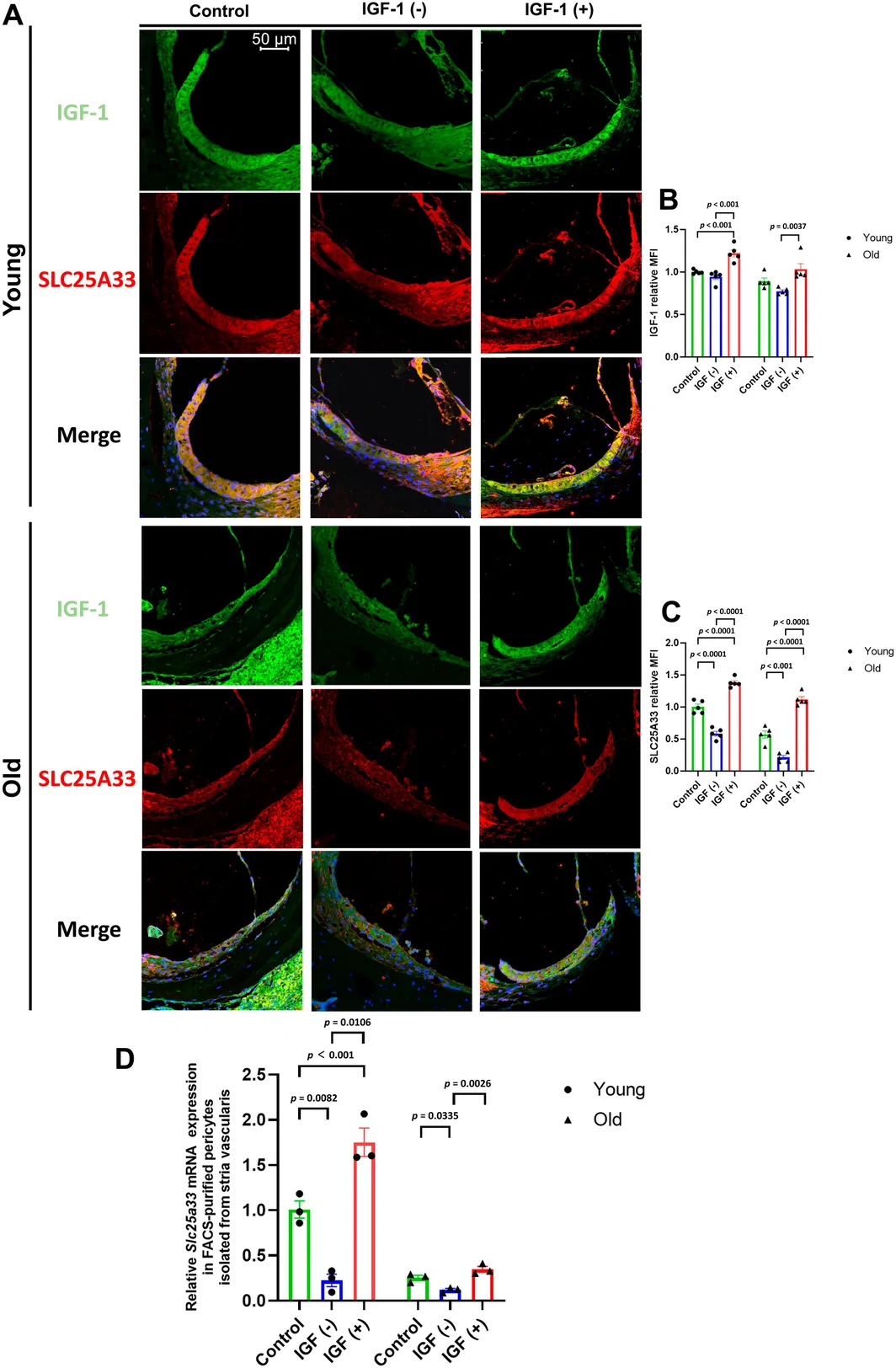
IGF‐1 upregulation promotes SLC25A33 expression in the stria vascularis of aged mice. (A) Immunofluorescence images show IGF‐1 (green) and SLC25A33 (red) expression in the stria vascularis. (B, C) Quantitative analysis reveals that exogenous IGF‐1 supplementation increases both IGF‐1 and SLC25A33 fluorescence intensity, while IGF‐1 inhibition further reduces their expression (*n* = 5, five typical sections from five individual mice were analyzed per group). (D) qPCR confirms exogenous IGF‐1 supplementation upregulates *Slc25a33* mRNA levels, whereas IGF‐1 inhibition decreases its expression (*n* = 3 mice). Control: wild‐type C57BL/6J mice without treatment; IGF‐1 (−): mice treated with NVP‐AEW541; IGF‐1 (+): mice treated with recombinant IGF‐1. Data are mean ± SEM.

### 3.6 SLC25A33 Modulates Pericyte Phenotype and TGF‐β Signaling In Vivo

Using AAV‐mediated gene delivery, we manipulated *Slc25a33* expression in the murine stria vascularis. After AAV injection via the round window membrane, altered expression of SLC25A33 was predominantly localized in the stria vascularis, and although moderate expression changes were also observed in endothelial cells and other cochlear cell types, the alterations were far more prominent in pericytes (Figure S5B–D).

Overexpression of *Slc25a33* significantly reduced α‐SMA levels, while its suppression increased α‐SMA immunoreactivity (Figure 5A,B). E‐cadherin expression followed an inverse pattern, being highest in the overexpression group and lowest in the knockdown group (Figure 5A,C). qPCR confirmed upregulation of *α‐Sma* and downregulation of *E‐cadherin* mRNA levels in the *Slc25a33*‐knockdown group (Figure 5D,E).

**FIGURE 5:**
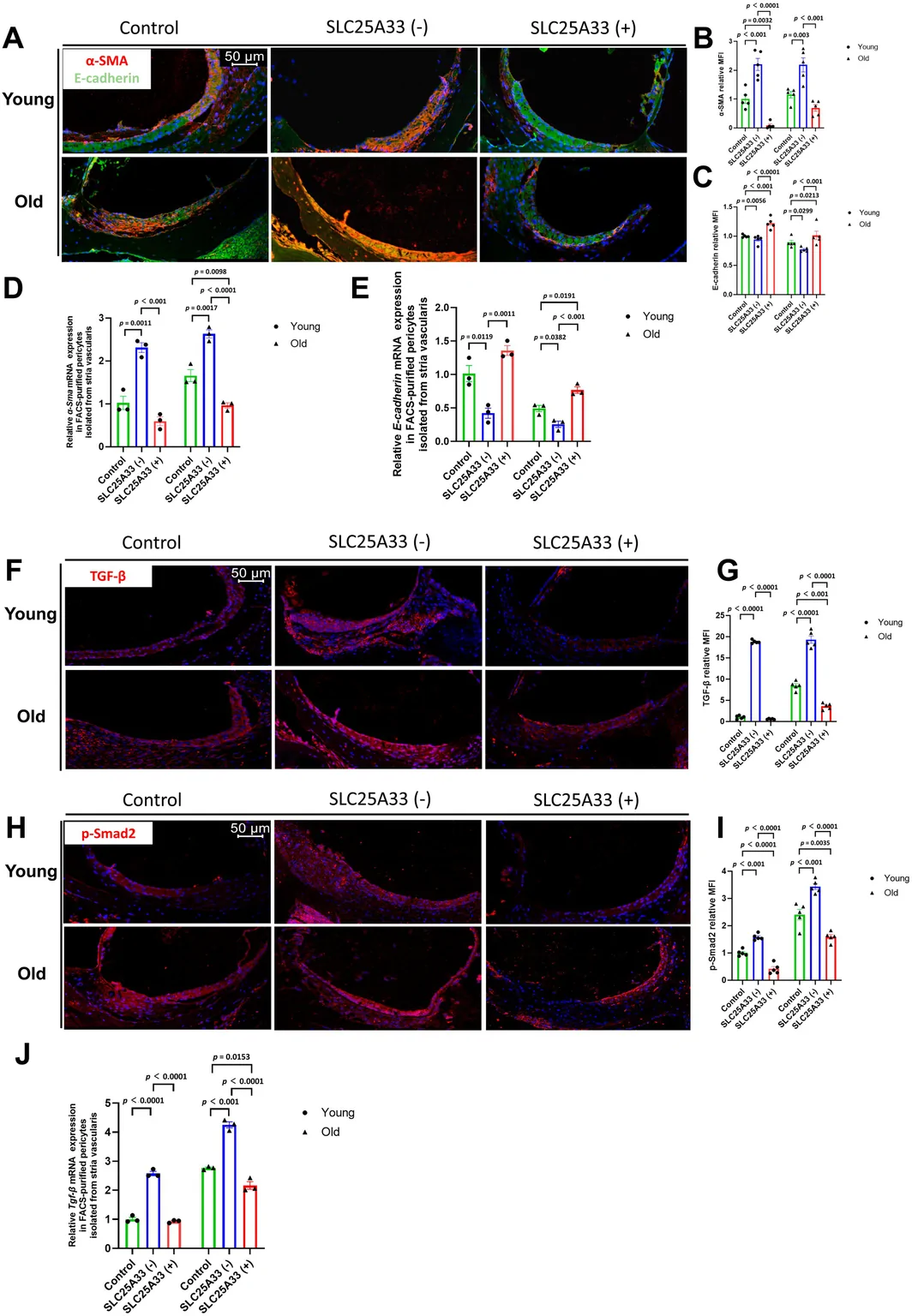
SLC25A33 modulates α‐SMA and E‐cadherin expression through the TGF‐β/Smad2 pathway in vivo. (A) Immunofluorescence staining shows α‐SMA (red) and E‐cadherin (green) expression in the stria vascularis. Nuclei are counterstained with DAPI (blue). (B, C) Quantification reveals increased α‐SMA and decreased E‐cadherin in the *Slc25a33*‐knockdown group (*n* = 5, five typical sections from five individual mice were analyzed per group). (D, E) qPCR confirms upregulation of *α‐Sma* and downregulation of *E‐cadherin* mRNA levels in the *Slc25a33*‐knockdown group (*n* = 3 mice). (F, G) TGF‐β and (H, I) p‐Smad2 immunoreactivity are enhanced in the knockdown group and reduced in the overexpression group (*n* = 5, five typical sections from five individual mice were analyzed per group). (J) qPCR confirms upregulation of *Tgf‐β* mRNA levels in the knockdown group and downregulation levels in the overexpression group (*n* = 3 mice). Control: wild‐type C57BL/6J mice without treatment; SLC25A33 (−): mice treated with AAV‐ie‐sh*Slc25a33*; SLC25A33 (+): mice treated with AAV‐ie‐*Slc25a33*‐OE. Data are mean ± SEM.

Consistent with in vitro results, TGF‐β and p‐Smad2 levels were lowest in the *Slc25a33*‐overexpressing group and highest in the suppressed group (Figure 5F–I), and qPCR confirmed the results in the pericytes isolated from stria vascularis of young and aged mice (Figure 5J), demonstrating that SLC25A33 regulates the TGF‐β/Smad2 pathway in vivo. We additionally treated *Slc25a33*‐suppressed aged mice with the TGF‐β receptor inhibitor LY2109761. Inhibition of TGF‐β signaling increased Claudin‐5 and Occludin levels in the stria vascularis, indicating alleviated BLB disruption (Figure S7E).

### 3.7 IGF‐1 Deficiency Disrupts BLB Integrity and Hearing Function, While IGF‐1 Supplementation Rescues Both

TEM revealed ultrastructural alterations in the BLB. In aged mice with IGF‐1 inhibition, pericytes displayed abnormal morphology and loose associations with endothelial cells, accompanied by widened tight junctions (Figure 6A). In contrast, exogenous IGF‐1 administration preserved pericyte–endothelial interactions and restored tight junction integrity.

**FIGURE 6:**
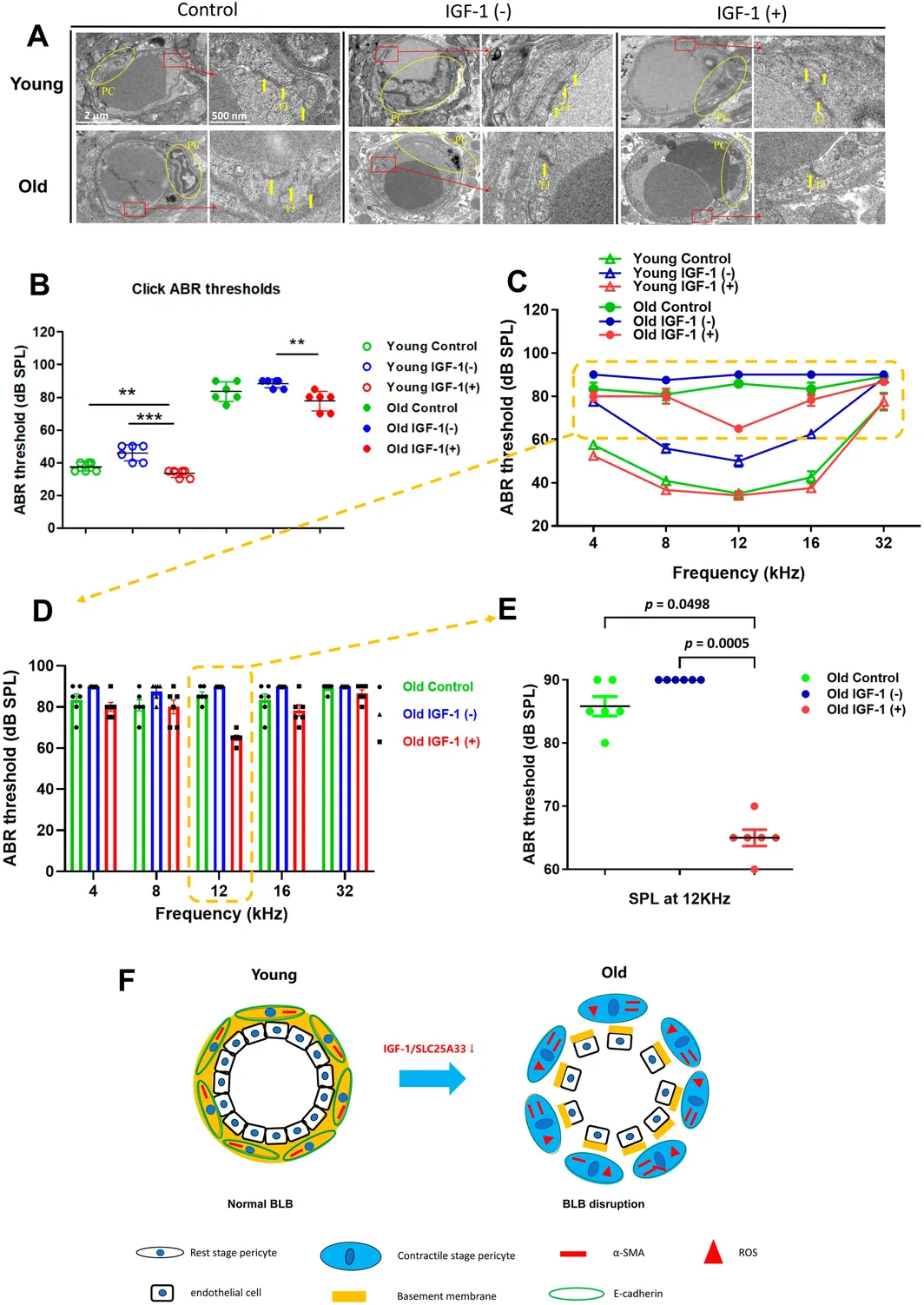
Exogenous IGF‐1 rescues blood‐labyrinth barrier integrity and improves hearing function in aged mice. (A) TEM images reveal ultrastructural changes in the stria vascularis. In the IGF‐1‐inhibited group, pericytes exhibit abnormal morphology, loose association with endothelial cells, and widened tight junctions (yellow arrows). These defects are ameliorated by exogenous IGF‐1 administration. (B) Click‐evoked ABR thresholds are significantly elevated in IGF‐1‐suppressed mice but partially rescued by IGF‐1 supplementation. (C–E) At 12 kHz, ABR thresholds are significantly lower in the IGF‐1‐treated group compared to control and suppressed groups, indicating improved auditory sensitivity. (F) Proposed model of IGF‐1/SLC25A33‐mediated regulation of pericyte phenotype and BLB integrity in age‐related hearing loss. Under physiological conditions, pericytes maintain a quiescent state characterized by low α‐SMA and high E‐cadherin expression, contributing to BLB stability. With aging, declining IGF‐1 levels lead to reduced SLC25A33 expression, resulting in mtROS accumulation and activation of the TGF‐β/Smad2 signaling pathway. This drives pericyte transition toward a contractile, pro‐fibrotic phenotype (high α‐SMA, low E‐cadherin), disrupting BLB integrity and promoting cochlear dysfunction. Targeting this axis may offer therapeutic potential for ARHL. Control: wild‐type C57BL/6J mice without treatment; IGF‐1 (−): mice treated with NVP‐AEW541; IGF‐1 (+): mice treated with recombinant IGF‐1. Data are mean ± SEM; *n* = 6 mice.

Functionally, IGF‐1 suppression exacerbated hearing loss in aged mice. Notably, IGF‐1 supplementation significantly lowered click‐evoked ABR thresholds compared to the suppressed group (Figure 6B). At 12 kHz, ABR thresholds were significantly improved in the IGF‐1‐treated group relative to both control and suppressed groups (Figure 6C–E), demonstrating functional recovery of auditory sensitivity.

## 4 Discussion

### 4.1 A Novel IGF‐1/SLC25A33–TGF‐β Axis Drives Pericyte Dysfunction in Age‐Related Hearing Loss

ARHL, the third most prevalent chronic condition in older adults, profoundly impacts quality of life by impairing communication, promoting social isolation, and accelerating cognitive decline (Dalton et al. 2003; Ege et al. 2024; Someya and Kim 2021). Despite its high prevalence, the molecular mechanisms underlying cochlear aging remain incompletely understood. In this study, we identify a previously unrecognized pathway in which age‐related IGF‐1 deficiency downregulates the mitochondrial carrier SLC25A33 in stria vascularis pericytes, leading to mtROS accumulation, activation of the TGF‐β/Smad2 signaling cascade, and subsequent pericyte phenotypic switching—ultimately resulting in BLB disruption and hearing impairment (Figure 6F). These findings establish the IGF‐1/SLC25A33–TGF‐β/Smad2 axis as a key regulator of microvascular integrity in the aging cochlea.

IGF‐1 is essential for inner ear development and lifelong auditory homeostasis, with roles in spiral ganglion neuron survival, hair cell protection, and strial function (Camarero et al. 2001; Camarero et al. 2002). SLC25A33 is an IGF‐1/IGF‐1R‐responsive mitochondrial nucleotide carrier implicated in OXPHOS maintenance and redox balance (Lyons et al. 2017). Suppression of SLC25A33 leads to the release of ROS, which results in increased mitochondrial biogenesis and an altered cellular differentiation programme (EMT). In contrast, overexpression of SLC25A33 suppresses ROS and EMT (Favre et al. 2010). Our transcriptomic analysis revealed coordinated downregulation of both *Igf1* and *Slc25a33* in pericytes isolated from aged stria vascularis, a finding corroborated by qPCR and immunofluorescence showing reduced protein expression in old mice with hearing loss. The above results also indicated that the change in IGF‐1/SLC25A33 expression was predominantly localized to pericytes. In addition, we validated this expression pattern in an independent mouse strain (CBA/CaJ), verifying that our findings were generalizable across strains (Figure S8).

The BLB, a critical component of the stria vascularis, maintains cochlear ion homeostasis and metabolic supply. Its dysfunction has been increasingly linked to ARHL pathogenesis (Shi 2011; Zhang et al. 2021). Pericytes are pivotal in BLB stabilization through physical coverage and paracrine signaling. Here, we demonstrate that suppression of SLC25A33 impairs mitochondrial function, leading to significant mtROS accumulation—a known driver of cellular stress and phenotypic transition. Importantly, SLC25A33‐deficient pericytes exhibited upregulated α‐SMA and downregulated E‐cadherin, hallmarks of a contractile, pro‐fibrotic phenotype. This shift mirrors the “pericyte‐to‐myofibroblast transition” observed in other microvascular beds, such as the blood–brain barrier, where elevated α‐SMA compromises vascular stability (Yao et al. 2014). Similarly, ROS‐induced EMT‐like transitions have been reported in various tissues (Xu et al. 2019), supporting our conclusion that SLC25A33 loss promotes pericyte activation via oxidative stress.

We further show that the TGF‐β/Smad2 pathway mediates this phenotypic switch. TGF‐β is a master regulator of fibrosis and mesenchymal differentiation, capable of directly inducing α‐SMA expression (Fernandez and Eickelberg 2012; Xu et al. 2019). Prior studies indicate that ROS can activate TGF‐β signaling in hepatocytes and renal cells (Sun et al. 2017), forming a feed‐forward loop between oxidative stress and fibrotic signaling. In our model, transcriptomic enrichment and functional assays confirm TGF‐β pathway activation upon SLC25A33 knockdown. Specifically, we observe increased TGF‐β expression and Smad2 phosphorylation, without changes in Smad3 or Smad4, highlighting selective engagement of Smad2, which may confer pathway specificity in pericyte regulation.

In vivo validation strengthens our mechanistic model. Modulation of IGF‐1 or SLC25A33 in aged mice recapitulated the in vitro findings: suppression led to p‐Smad2 upregulation, α‐SMA induction, E‐cadherin loss, and ultrastructural BLB damage, including widened tight junctions and pericyte detachment. Conversely, exogenous IGF‐1 supplementation restored SLC25A33 expression, attenuated TGF‐β signaling, preserved BLB integrity, and significantly improved auditory function, as evidenced by lower ABR thresholds. This partial but significant rescue underscores the therapeutic potential of targeting the IGF‐1/SLC25A33 axis in ARHL.

### 4.2 Limitations and Future Directions

While our data support a central role for pericyte dysfunction in ARHL, several questions remain. First, whether IGF‐1 acts directly on pericytes via IGF‐1R or indirectly through autocrine/paracrine networks requires further investigation using cell‐specific knockout models. Second, although we focused on SLC25A33, IGF‐1 likely regulates additional mitochondrial and cytoprotective genes that may contribute to BLB stability. Third, species differences and brain vs. cochlear pericyte identity may affect result interpretation, which requires further validation in future studies.

## 5 Conclusion

In summary, we uncover a novel IGF‐1‐SLC25A33‐mtROS‐TGF‐β/Smad2‐pericyte activation axis that links hormonal signaling to microvascular degeneration in the aging cochlea. Our work not only advances the understanding of ARHL pathogenesis but also identifies SLC25A33 as a potential biomarker and therapeutic target for preserving hearing in aging populations. Targeting pericyte‐mediated BLB dysfunction may represent a new frontier in the treatment of sensorineural hearing loss.

## Author Contributions

**Rui Xu:** data curation, formal analysis, writing original draft. **Jia Tang:** data curation, formal analysis, writing original draft. **Huan Lin:** data curation, formal analysis, writing original draft. **Qi Li:** data curation, writing – review and editing. **Yajin Feng:** data curation, writing – review and editing. **Xiaorong Zhang:** data curation, writing – review and editing. **Xiaohong Hu:** data curation, writing – review and editing. **Jiacai Yang:** data curation, writing – review and editing. **Yuhong Li:** conceptualization, data curation, formal analysis, writing original draft. **Yang Bai:** conceptualization, data curation, formal analysis, writing original draft.

## Funding

This work was supported by National Natural Science Foundation for Young Scientists of China (No. 82201298), Miao Pu project of Army Medical University (2017R016).

## Ethics Statement

All animal procedures were approved by the Animal Ethics Committee of the Army Medical University (AMUWEC20224078) and conducted in accordance with the ARRIVE guidelines.

## Conflicts of Interest

The authors declare no conflicts of interest.

## Supporting information


**Table S1:** Sequences of primers used in pericytes experiments.
**Table S2:** Details of mice used in the animal experiments.
**Tabel S3**. Statistics of differentially expressed genes.
**Table S4:** List of partial DEGs: young versus old (IGF‐1 included, ranked by *p*. adjust).
**Table S5:** List of partial DEGs: young versus old (SLC25A33 included, ranked by *p*. adjust).
**Figure S1:** (A) Representative flow cytometry plots illustrating the gating strategy for isolating pericytes from microdissected stria vascularis tissues. (B) *Pdgfrb* mRNA expression was measured by qPCR for post‐sort validation.
**Figure S2:** Identification of primary pericytes isolated from postnatal (P12–P16) C57BL/6J mice. (A) Immunofluorescence staining for PDGFR‐β; (B) Flow cytometry identification.
**Figure S3:** (A) Immunofluorescence staining of IGF‐1, IGF‐1R and SLC25A33 in cultured primary pericytes isolated from the stria vascularis of postnatal (P12–P16) mice. Nuclei are counterstained with DAPI (blue). (B) Quantitative PCR analysis of FACS‐sorted pericytes isolated from the stria vascularis of young and aged mice revealed no significant difference in *Igf1r* mRNA levels between the two groups.
**Figure S4:** (A) Representative Western blot images of SLC25A33. (B) Changes of IGF‐1 expression did not significantly alter SLC25A33 protein expression levels in endothelial cells.
**Figure S5:** (A) The knockdown efficiency of LV‐*Slc25a33*‐shRNA and overexpression efficiency of LV‐*Slc25a33*‐OE were validated by Western blot. (B) Immunofluorescence staining validated the AAV‐injected mouse model as well as verified the upregulation and downregulation of SLC25A33 expression in pericytes. In AAV‐injected mice, SLC25A33 expression was significantly altered in (C) stria vascularis, especially in (D) pericytes. (E) AAV‐ie‐sh*Slc25a33* induced substantial hearing loss in young mice. (F) In aged animals, ABR thresholds at 4 and 12 kHz were notably improved in the *Slc25a33* overexpression group, compared with the suppressed group. Data are mean ± SEM; *n* = 6 mice.
**Figure S6:** (A) MitoSOX Red‐based flow cytometry demonstrated diminished mtROS generation in MitoTEMPO‐treated *Slc25a33*‐suppressed HBVPs when compared with untreated *Slc25a33*‐suppressed HBVPs and H₂O₂‐exposed *Slc25a33*‐suppressed HBVPs. (B) Quantification of mtROS fluorescence intensity confirmed this decrease. (C) Representative Western blot of TGF‐β, p‐Smad2 and α‐SMA protein expression. Densitometric quantification demonstrated reduced (D) TGF‐β, (E) p‐Smad2 and (F) α‐SMA protein in MitoTEMPO‐treated *Slc25a33*‐suppressed HBVPs.
**Figure S7:** Manipulation of the TGF‐β pathway in SLC25A33‐deficient pericytes and AAV‐ie‐sh*Slc25a33* treated old mice using TGF‐β receptor inhibitor (LY2109761). (A) Representative Western blot images of p‐Smad2, α‐SMA and E‐cadherin. The results demonstrated that inhibition of the TGF‐β signaling pathway in SLC25A33‐ deficient pericytes significantly downregulated the protein levels of (B) p‐Smad2 and (C) α‐SMA, while increasing (D) E‐cadherin expression. (E) The AAV‐sh*Slc25a33*‐injected aged mice were treated with the TGF‑β receptor inhibitor LY2109761. Inhibition of TGF‑β signaling increased Claudin‑5 and Occludin levels in the stria vascularis, indicating alleviated BLB disruption.
**Figure S8:** Validation of IGF‐1/SLC25A33 expression pattern in an independent mouse strain (CBA/CaJ). (A) Representative western blot images for IGF‑1 and SLC25A33 in stria vascularis collected from young (3‑month‑old) and old (30‑month‑old) CBA/CaJ mice. Western blot analysis of isolated stria vascularis tissues revealed significantly reduced (B) IGF‐1 and (C) SLC25A33 protein levels in aged animals. The results verified that our findings were generalizable across strains.

## Data Availability

Data generated or analyzed during this study are available from the corresponding author upon reasonable request.
