# Supplementary material for: IGF‐1/SLC25A33 Maintains Blood‐Labyrinth Barrier Integrity by Suppressing TGF‐β/Smad2‐Mediated Pericyte Activation in Age‐Related Hearing Loss
Source: Aging Cell. 2026 Sep 29;25(10):e70712. doi: 10.1111/acel.70712 (PMC13621939; doi:10.1111/acel.70712)
Supplement: Supplementary file 1 [file ACEL-25-e70712-s001.docx]

***Supplementary materials***

**IGF-1/SLC25A33 Maintains Blood-Labyrinth Barrier Integrity by Suppressing TGF-β/Smad2-Mediated Pericyte Activation in Age-Related Hearing Loss**

Rui Xu ^1,2,#^, Jia Tang ^1^^,3,#^, Huan Lin ^1,#^, Qi Li ^1^, Yajin Feng ^1^, Xiaorong Zhang ^4^, Xiaohong Hu ^4^, Jiacai Yang ^4^, Yuhong Li ^3,*^, Yang Bai ^1,*^

^1^ Department of Otolaryngology, The First Affiliated Hospital, Army Medical University, Gaotanyan Zheng Street, Shapingba District, Chongqing 400038, China.

^2^ Department of Neurology, The Second Affiliated Hospital, Army Medical University, Xinqiao Zheng Street, Shapingba District, Chongqing 400037, China.

^3^ Department of Cell Biology, Army Medical University, Chongqing 400038, China.

^4^ Institute of Burn Research, The First Affiliated Hospital, Army Medical University, Gaotanyan Zheng Street, Shapingba District, Chongqing 400038, China.

^#^ These authors contributed equally to this work.

*** Correspondence:**

Yuhong Li: liyuhong@tmmu.edu.cn, Tel: +86 2368771373;

Yang Bai: baiyang-ent@tmmu.edu.cn, Tel: +86 2368766282.

**Table S1. Sequences of Primers Used in Pericytes Experiments**

|  | Forward primer | Reverse primer |
| --- | --- | --- |
| *Igf-1* | 5'-GCTGAGTTGGTG  ATGCTGGT-3' | 5'-CAGTACATCTCCA  GTCTCCTCAG-3' |
| *Igf1r* | 5′-GGCTGCTGA AGTTGG  AAGATG-3′ | 5′-GGTGGTGTCTGTGTC TTGTCTG-3′ |
| *Slc25a33* | 5ʹ- AAAGTTCCTCTGG  CTTCTTTGGGA-3ʹ | 5ʹ- GAGCAACAGTCCT  CGGTAGAA-3ʹ |
| *Pdgfrb* | 5′-ATCTTCCTGGTGCT  GCTGTT-3′ | 5′-GTGGTGAAGAGGTCG  GTGTT-3′ |
| *α-Sma* | 5ʹ- CACGATGTAC  CCTGGGATCG-3ʹ | 5ʹ- GCCGATCCAC  ACCGAGTATT-3ʹ |
| *E-cadherin* | 5ʹ- TAGAGAACGC  ATTGCCACATACA-3ʹ | 5ʹ- GAATTCCGGC  TTGTTGTCATTCT-3ʹ |
| *Tgf-β1* | 5ʹ- TACAGCAACAAT  TCTGGCGATA -3ʹ | 5ʹ- CAGTGTGTTATC  CCTGCTGTCAC -3ʹ |
| *Smad2* | 5ʹ-ACACACCAGATC  CTAACAGAAC-3ʹ | 5ʹ-GGAGGTGGCGTT  TCTGGATATA-3ʹ |
| *Smad3* | 5ʹ-GAACACAGTGAC  ACAGATGAA-3ʹ | 5ʹ-TGGCTGCAGGTC  ACAGTTATTAT-3ʹ |
| *Smad4* | 5ʹ-GGACTGCACCAT  ACACACCTAAT-3ʹ | 5ʹ-ACAGTAACATAG  GGCAGCTTGA-3ʹ |

Table S2. **Details of Mice Used in the Animal Experiments**

| Characteristics | Young  (n = 70) | Old  (n = 67) | *P* |
| --- | --- | --- | --- |
| Age, mos — Mean ± SD | 3.5 ± 0.5 | 17.49 ± 0.5 | < 0.01 |
| Male sex — no. (%) | 36 (51.4) | 34 (50.7) | 0.99 |
| Weight,g —Mean ± SD | 23.7 ± 2.6 | 29.0 ± 2.8 | < 0.01 |
| Hearing loss — no. (%) | 0 (0) | 57 (85.0) | < 0.01 |

**Tabel S3. Statistics of Differentially Expressed Genes**

| **Compare** | **All** | **Up** | **Down** | **Threshold** |
| --- | --- | --- | --- | --- |
| Young_vs_Old | 9239 | 4811 | 4428 | \|log2FoldChange\|>1&pvalue<0.05 |

**Table S4. List of Partial DEGs: Young vs Old (IGF-1 Included, ranked by *p.adjust*)**

**Table S5. List of Partial DEGs: Young vs Old (SLC25A33 Included, ranked by *p.adjust*)**

**Figure S1.** (A) Representative flow cytometry plots illustrating the gating strategy for isolating pericytes from microdissected stria vascularis tissues. (B) *Pdgfrb* mRNA expression was measured by qPCR for post-sort validation.

**Figure S2.** Identification of primary pericytes isolated from postnatal (P12–P16) C57BL/6J mice. (A) Immunofluorescence staining for PDGFR-β; (B) Flow cytometry identification.

**Figure S3.** (A) Immunofluorescence staining of IGF-1, IGF-1R and SLC25A33 in cultured primary pericytes isolated from the stria vascularis of postnatal (P12–P16) mice. Nuclei are counterstained with DAPI (blue). (B) Quantitative PCR analysis of FACS-sorted pericytes isolated from the stria vascularis of young and aged mice revealed no significant difference in *Igf1r* mRNA levels between the two groups.

**Figure S4. (A)** Representative Western blot images of SLC25A33. (B) Changes of IGF-1 expression did not significantly alter SLC25A33 protein expression levels in endothelial cells.

**Figure S5.** (A) The knockdown efficiency of LV-*Slc25a33*-shRNA and overexpression efficiency of LV-*Slc25a33*-OE were validated by Western blot. (B) Immunofluorescence staining validated the AAV-injected mouse model as well as verified the upregulation and downregulation of SLC25A33 expression in pericytes. In AAV-injected mice, SLC25A33 expression was significantly altered in (C) stria vascularis, especially in (D) pericytes. (E) AAV-ie-sh*Slc25a33* induced substantial hearing loss in young mice. (F) In aged animals, ABR thresholds at 4 and 12 kHz were notably improved in the *Slc25a33* overexpression group, compared with the suppressed group. Data are mean ± SEM; *n* = 6 mice.

**Figure S6.** (A) MitoSOX™ Red-based flow cytometry demonstrated diminished mtROS generation in MitoTEMPO-treated *Slc25a33*-suppressed HBVPs when compared with untreated *Slc25a33*-suppressed HBVPs and H₂O₂-exposed *Slc25a33*-suppressed HBVPs. (B) Quantification of mtROS fluorescence intensity confirmed this decrease. (C) Representative Western blot of TGF-β, p-Smad2 and α-SMA protein expression. Densitometric quantification demonstrated reduced (D) TGF-β, (E) p-Smad2 and (F) α-SMA protein in MitoTEMPO-treated *Slc25a33*-suppressed HBVPs.

**Figure S7.** Manipulation of the TGF-β pathway in SLC25A33-deficient pericytes and AAV-ie-sh*Slc25a33* treated old mice using TGF-β receptor inhibitor (LY2109761). **(A)** Representative Western blot images of p-Smad2, α-SMA and E-cadherin. The results demonstrated that inhibition of the TGF-β signaling pathway in SLC25A33- deficient pericytes significantly downregulated the protein levels of (B) p-Smad2 and (C) α-SMA, while increasing (D) E-cadherin expression. (E) The AAV-sh*Slc25a33*-injected aged mice were treated with the TGF‑β receptor inhibitor LY2109761. Inhibition of TGF‑β signaling increased Claudin‑5 and Occludin levels in the stria vascularis, indicating alleviated BLB disruption.

**Figure S8.** Validation of IGF-1/SLC25A33 expression pattern in an independent mouse strain (CBA/CaJ). (A) Representative western blot images for IGF‑1 and SLC25A33 in stria vascularis collected from young (3‑month‑old) and old (30‑month‑old) CBA/CaJ mice. Western blot analysis of isolated stria vascularis tissues revealed significantly reduced (B) IGF-1 and (C) SLC25A33 protein levels in aged animals. The results verified that our findings were generalizable across strains.

**Supplementary Methods**

**Lentiviral Transduction for SLC25A33 Modulation**

The lentiviral vector for SLC25A33 overexpression (LV-*Slc25a33*-OE, product No. LVP0728-1-2, titer: 3.0 × 10⁸ TU/mL) was constructed with the backbone plasmid pLV4ltr-PGK-ZsGreen1(2A)Hyg-CMV-c3×FLAG. This vector harbors the full-length coding sequence of human SLC25A33 driven by the CMV promoter. The sequence below is the coding region of human SLC25A33, which contains the Kozak consensus sequence (GCCACC) at the 5′ end and the stop codon (TAA) at the 3′ end. The sequence was provided by the manufacturer and verified by Sanger sequencing.

>SLC25A33_insert (984 bp) Nucleotide sequence (5′ → 3′)

GCCACCATGGCGACGGGCGGCCAGCAGAAGGAGAACACGCTGCTTCACCTCTTCGCCGGCGGGTGTGGAGGCACAGTTGGTGCTATTTTCACTTGTCCACTAGAAGTCATTAAGACACGGTTGCAGTCTTCAAGATTAGCTCTCCGGACAGTCTACTATCCTCAGGTTCATCTGGGGACCATTAGTGGAGCTGGAATGGTGAGACCAACATCCGTGACACCTGGACTCTTTCAGGTTCTGAAGTCGATCTTGGAGAAAGAGGGACCAAAGTCACTTTTTAGAGGCTTGGGTCCAAATTTGGTTGGAGTTGCACCATCAAGGGCTGTATACTTTGCATGTTACTCCAAAGCCAAAGAGCAATTTAATGGCATTTTCGTGCCTAACAGCAATATTGTGCATATTTTCTCAGCTGGCTCTGCAGCTTTTATCACAAATTCCTTAATGAATCCTATATGGATGGTTAAAACCCGAATGCAGCTAGAACAGAAAGTGAGGGGCTCTAAGCAGATGAATACACTCCAGTGTGCTCGTTACGTTTACCAGACCGAAGGCATTCGTGGCTTCTATAGAGGATTAACTGCCTCGTATGCTGGAATTTCCGAAACTATAATCTGCTTTGCTATTTATGAAAGTTTAAAGAAGTATCTGAAAGAAGCTCCATTAGCCTCTTCTGCAAATGGGACTGAGAAAAATTCCACAAGTTTTTTTGGACTTATGGCAGCTGCTGCTCTTTCTAAGGGCTGTGCCTCCTGCATTGCTTATCCACACGAAGTCATAAGGACGAGGCTCCGGGAAGAGGGCACCAAGTACAAGTCTTTTGTCCAGACGGCGCGCCTGGTGTTCCGGGAAGAAGGCTACCTTGCCTTTTATAGAGGACTGTTTGCCCAGCTTATCCGGCAGATCCCAAATACTGCCATTGTGTTGTCTACTTATGAGTTAATTGTGTACCTGTTAGAAGACCGTACTCAGTAA

The lentiviral vector for *Slc25a33* knockdown (LV-*Slc25a33*-shRNA, product No. 20240318J4-3, titer: 2.0 ×10⁸ TU/mL) expresses a shRNA with the targeting sequence of 5′-GCTTATCCACACGAAGTCATA-3′ under the control of the U6 promoter.

All lentiviral particles were purchased from TsingkeBiotechnologyCo., Ltd. (Beijing, China).
